# Supplementary material for: The Pan-Cancer Multi-Omics Landscape of FOXO Family Relevant to Clinical Outcome and Drug Resistance
Source: Int J Mol Sci. 2022 Dec 9;23(24):15647. doi: 10.3390/ijms232415647 (PMC9778770; doi:10.3390/ijms232415647)
Supplement: Supplementary file 1 [file ijms-23-15647-s001.zip › ijms-2027212-supplementary.pdf]

Supplementary Materials:

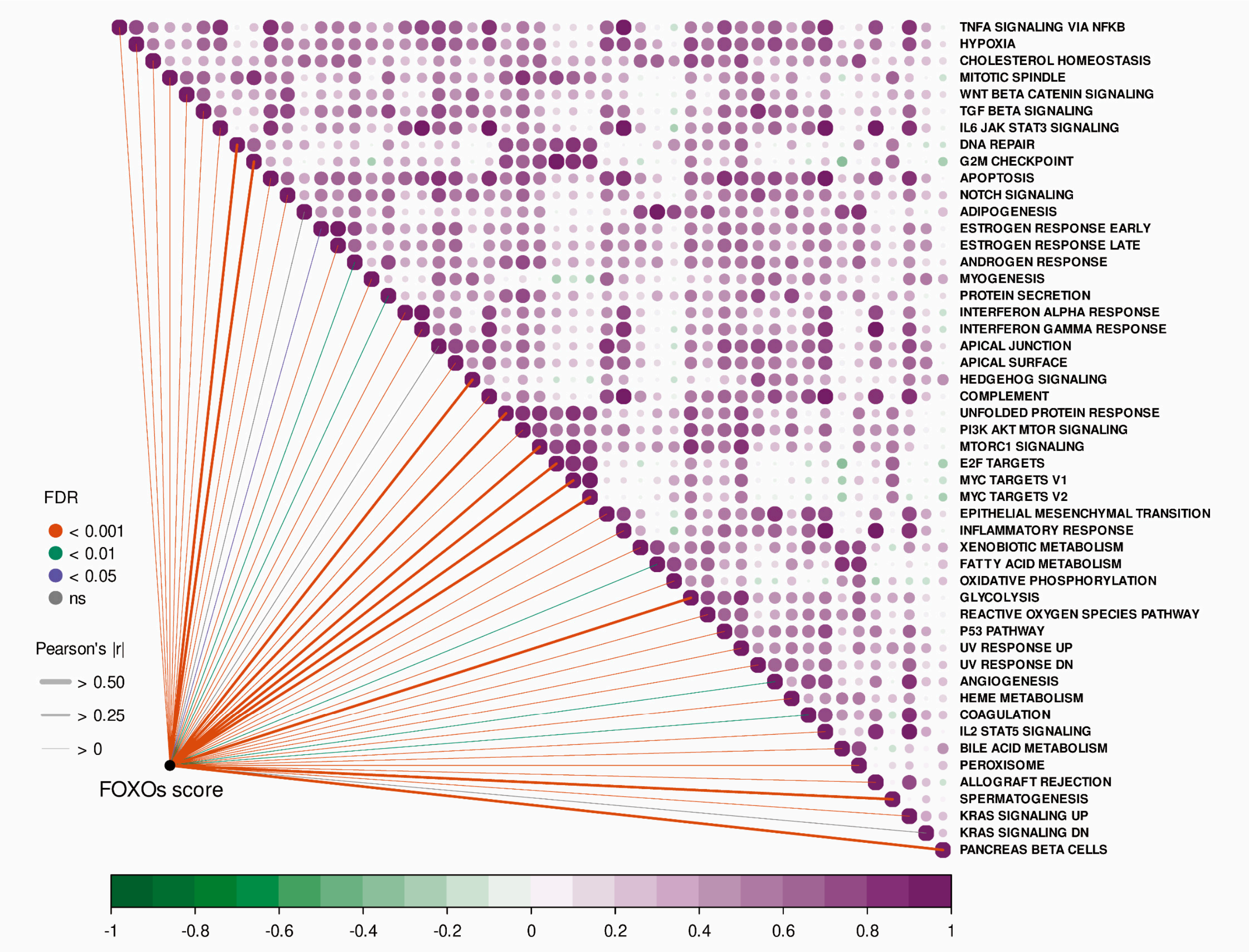

Supplementary Figure S1. The general correlations between FOXOs score and hallmark pathways in the TCGA pan-cancer cohort (FDR, false discovery rate).

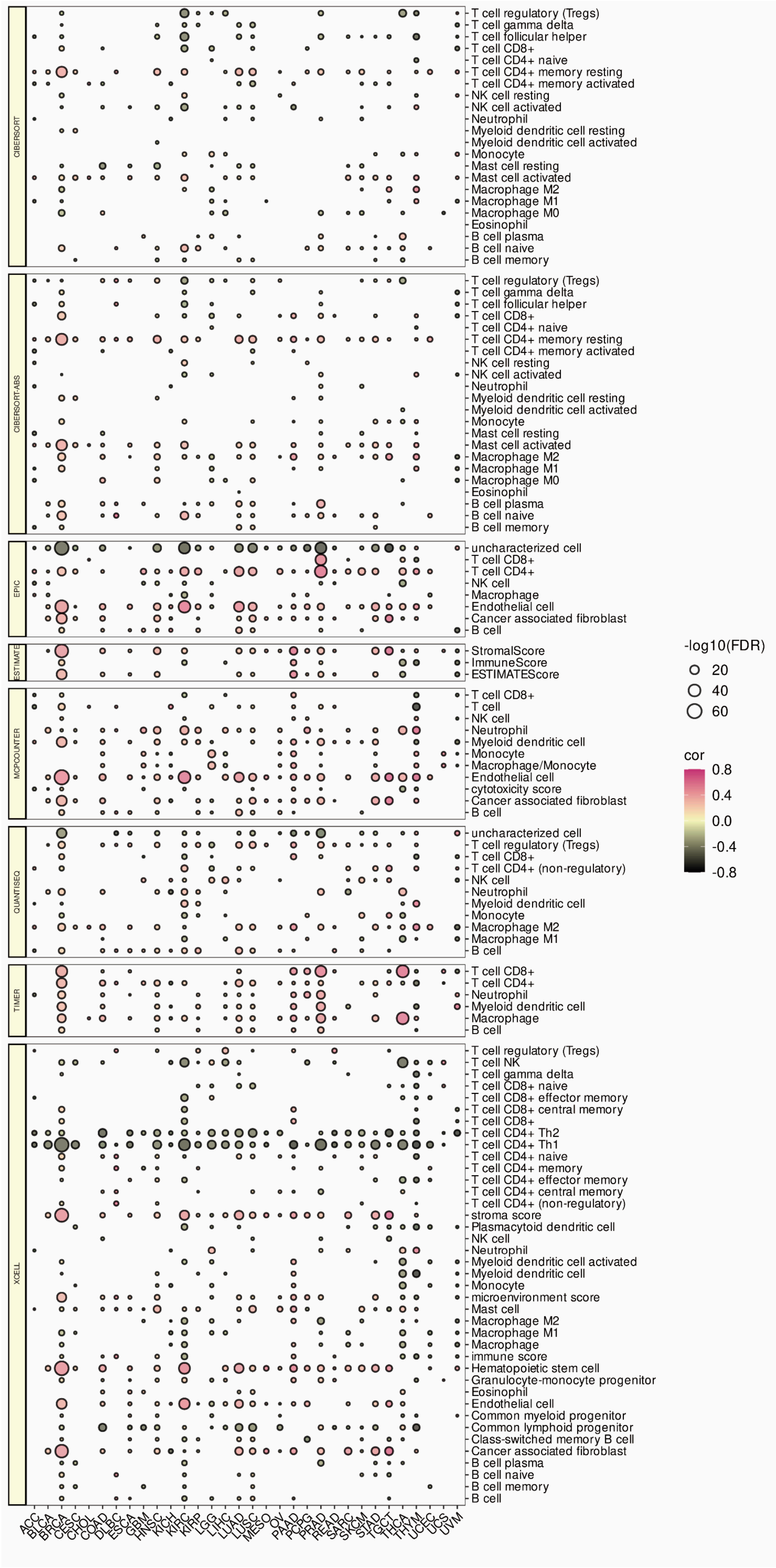

Supplementary Figure S2. Bubble plot of the correlations between FOXOs score and tumor microenvironment scores calculated by different algorithms in each tumor type (FDR, false discovery rate).
